# Stereospecific Antifungal Activity of Strigolactone Analogues Against *Botrytis cinerea* and *Sclerotinia sclerotiorum*

**DOI:** 10.3390/jof12050359

**Published:** 2026-05-13

**Authors:** Pingliang Huang, Ruifeng Yao, Li Chen

**Affiliations:** 1 State Key Laboratory of Chemo and Biosensing, Hunan Provincial Key Laboratory of Plant Functional Genomics and Developmental Regulation, College of Biology, Longping Agricultural College, Hunan University, Changsha 410082, China; hpl2021@hnu.edu.cn; 2 Yuelushan Laboratory, Changsha 410082, China; 3 Hunan Research Center of the Basic Discipline for Cell Signaling, College of Biology, Hunan University, Changsha 410082, China

**Keywords:** strigolactone analogues, stereospecificity, antifungal activity, *Botrytis cinerea*, *Sclerotinia sclerotiorum*

## Abstract

Plant hormones and their synthetic analogueues offer sustainable alternatives for crop protection, yet the direct antifungal activity of strigolactone (SL) and its analogues against necrotrophic pathogens remain largely unexplored. Here, we screened eight phytohormones and related analogues for treatments of *Botrytis cinerea* and identified the SL analogue *rac*-GR24 (racemic GR24) as a concentration-dependent growth inhibitor active at low micromolar concentrations. Given the stereochemical complexity of SLs and their analogues, we evaluated multiple enantiopure isomers and found that *ent*-5DS and GR24*^ent^*^-5DS^, which differ in configuration from natural SLs, exhibited the strongest inhibitory activity. This stereospecific response was further validated using another filamentous fungus, *Sclerotinia sclerotiorum*, which displayed an identical susceptibility profile. Combinatorial treatments with enantiopure isomers and double-concentration *rac*-GR24 revealed that the antifungal effect of the racemate is primarily attributable to the GR24*^ent^*^-5DS^ enantiomer, whereas the opposite enantiomer GR24^5DS^ is almost inactive. Collectively, our findings uncover a stereospecific response in fungal pathogens, demonstrating that *B. cinerea* and *S. sclerotiorum* respond to exogenous SL analogues in a chirally selective manner. This work establishes a stereochemically defined framework for developing enantioselective fungicidal agents with potential applications in sustainable agriculture.

## 1. Introduction

A wide range of filamentous fungi are devastating pathogens of major crops. Among them are *Botrytis cinerea* and *Sclerotinia sclerotiorum*, two typical necrotrophic fungi that cause severe yield losses. *B. cinerea* causes annual global economic losses estimated at 10 billion to 100 billion across more than 1400 plant species, with particularly severe impacts on grapevine, strawberry, tomato, and ornamentals. *S. sclerotiorum* affects over 400 plant species worldwide, with yield losses ranging from 20 to 60% in susceptible crops such as oilseed rape, soybean, and sunflower, annual economic losses are estimated at hundreds of millions of dollars [1,2,3,4,5]. The fungus affects crops throughout the growth cycle, from seedlings to maturity [6]. Owing to the difficulty of its control, *B. cinerea* causes severe yield losses and substantial economic damage worldwide. *B. cinerea* propagates primarily asexually via the production of abundant conidia and the formation of sclerotia, the latter of which also enables sexual reproduction following fertilization by microconidia [7,8,9,10]. Although the fungus is capable of producing ascospores, conidia serve as the main propagules and represent the primary source of inoculum, being widely distributed in the air [11,12,13]. Conidia land on plant surfaces, germinate to form short germ tubes, and penetrate directly through the leaf cuticle. Infection can also occur from established hyphae, which form multicellular infection cushions. Following penetration, epidermal and cortical cells undergo sequential cell death, allowing the establishment of primary infection [14,15,16,17,18,19].

Current management strategies for resistance of filamentous fungi depend heavily on chemical fungicides; however, their overuse has precipitated a marked increase in fungicide resistance, compromising disease control efficacy [20,21]. Consequently, there is an urgent need for sustainable alternatives. The development and application of plant-derived fungicides have become a central pillar of integrated management strategies against grey mould caused by *B. cinerea.* Such fungicides are primarily sourced from plant secondary metabolites, whose active ingredients are extracted and purified to yield efficacious pathogen inhibitors [22,23,24]. Concurrently, recent studies have highlighted the antifungal potential of alternative approaches, such as chitosan–zinc oxide nanocomposites, underscoring the growing diversity of emerging strategies for plant protection [25]. In studies on disease resistance mechanisms mediated by plant endogenous compounds, exogenous application of salicylic acid has been shown to significantly enhance vascular bundle lignification in Arabidopsis thaliana. This effect restricts pathogen infection by inducing lignin synthesis [26,27,28,29]. Furthermore, both jasmonic acid (JA) and methyl jasmonate (MeJA) enhance tomato resistance against *B. cinerea*, whereas upregulation of repressive components in the JA signalling pathway reduces their ability to resist *B. cinerea* infection [23].

Strigolactones (SLs) are a class of plant hormones initially identified for their ability to induce germination of the parasitic plant *Striga* [30,31]. Based on their chemical structure, SLs are classified into canonical and non-canonical types. Canonical SLs contain a tricyclic lactone (ABC-ring) connected to a butenolide moiety (D-ring) via an enol ether bond. Non-canonical SLs lack the ABC-ring but retain the conserved enol ether bond and D-ring [32,33]. Subsequent studies have revealed that, in addition to regulating parasitic weed germination, SLs play multiple key physiological roles, including promoting arbuscular mycorrhizal fungal (AMF) symbiosis with host plant roots, modulating shoot branching, and mediating responses to abiotic stress [34,35,36,37,38]. Natural SLs comprise a family of over 20 compounds, which differ primarily in the methylation pattern of the cyclohexenyl A-ring or in the hydroxyl/acetyloxy substitutions on the A and B rings. Owing to the difficulty of obtaining sufficient quantities of natural SLs for physiological studies, various synthetic analogueues with simpler structures but retained biological activity have been developed [39,40]. Among these, the GR (germination releaser) series, including *rac*-GR24 (racemic GR24), GR7, and GR5, are artificially synthesized strigol analogues. *rac*-GR24, a commercially available 5-deoxystrigol mimetic, exhibits comparable activity and stability with natural SLs and has become the most widely used SL analog in SL research, particularly for studies on plant development and rhizosphere interactions [41,42,43].

In addition to regulating plant growth and development, SLs mediate various symbiotic interactions with microorganisms. Upon release into the soil, SLs are perceived by AMF, stimulating mitochondrial aerobic respiration, hyphal elongation, and spore germination, thereby promoting symbiosis establishment and enhancing host plant nutrient uptake and growth [44]. Beyond their effects on mycorrhizal fungi and parasitic plants, SLs also influence plant pathogenic fungi. Exogenous treatment with *rac*-GR24 has been shown to induce multiple responses in pathogenic fungi, including hyphal branching, altered growth, increased respiratory activity, ATP and NADPH production, mitosis, and effector gene expression [45,46,47]. Notably, fungal colony growth rate decreases in a *rac*-GR24 concentration-dependent manner. GR24 can also affect fungal pathogenicity of *Mucor* by restricting hyphal growth and inducing reactive oxygen species (ROS) production [10,48]. However, the precise conformational and stereochemical determinants required for fungal recognition of these small molecules remain to be elucidated.

Although accumulating evidence indicates that GR24 inhibits phytopathogenic fungi, it remains unclear whether filamentous fungi possess a stereoselective recognition mechanism for SL analogues. Specifically, the stereoisomeric configuration responsible for the observed antifungal activity has yet to be identified, and it is unknown whether such stereospecificity is conserved among distinct necrotrophic fungal species. Accordingly, this study aimed to screen a panel of plant hormones and their analogues to identify SL-based inhibitors active against *B. cinerea*, to evaluate the concentration-dependent response of rac-GR24 and systematically assess the antifungal activity of natural and synthetic SL stereoisomers, and to determine whether the observed stereospecificity extends to another necrotrophic pathogen, *S. sclerotiorum*. By elucidating the stereochemical determinants of antifungal activity, this study provides a theoretical foundation and practical reference for the development of enantioselective fungicides.

## 2. Materials and Methods

### 2.1. Fungal Strains and Culture Conditions

*Botrytis cinerea* wild-type strain B05.10 and *Sclerotinia sclerotiorum* were cultured at 22 °C in the dark on potato dextrose agar media (PDA; 200 g/L peeled potato, 20 g/L glucose, and 16 g/L agar). *B. cinerea* and *S. sclerotiorum* strains were kindly provided by Paul Tudzynski (University of Münster, Germany), Shiping Tian (Institute of Botany, Chinese Academy of Sciences), and the Hubei Academy of Agricultural Sciences. All small-molecule compounds, including the eight SL-related stereoisomers and the additional phytohormones and their analogues, were purchased from Olchemim company (Olomouc, Czech Republic) (https://www.olchemim.cz/Products.aspx?idc=1&idp=30, accessed on 5 May 2025) or Chiralix company (Nijmegen, The Netherlands) (https://www.chiralix.com/en/home, accessed on 5 May 2025).

### 2.2. The Poisoned Food Technique

For each experimental setup, the wild-type strain was pre-cultured on PDA at 22 °C in the dark for 48 h. Mycelial plugs (5 mm diameter) were excised from the actively growing peripheral zone of the colonies and transferred to PDA plates supplemented with the indicated compounds (preparation details are provided below). Plates were incubated at 22 °C in the dark. At specified time points, colony diameters were measured, and the colony area was calculated. Relative colony area (%) was determined as (treatment colony area/control colony area) × 100%.

### 2.3. Growth Detection of Botrytis cinerea Treated with Different Small Molecules

To assess the sensitivity of *B. cinerea* to different small molecules, stock solutions of *rac*-GR24 (a synthetic strigolactone analogue), D-OH (the hydrolyzed product of GR24 after receptor recognition, lacking SL activity [49,50,51], carba-GR24 (a non-hydrolyzable GR24 derivative, also inactive) [52], methyl jasmonate (MeJA) and jasmonic acid-Isoleucine (JA-Ile, two forms of the defence-related phytohormone jasmonic acid), coronatine (COR, a JA analogue) [23], abscisic acid (ABA, a phytohormone primarily involved in abiotic stress responses) [53], and indole-3-acetic acid (IAA, a major natural auxin regulating plant growth and development) [54] were first dissolved in DMSO to prepare stock solutions at concentrations of 10 mM or 20 mM, respectively. Each stock was added at 0.1% (*v*/*v*) to cooled PDA medium to achieve final working concentrations of 10 μM or 20 μM of the respective compound. PDA supplemented with 0.1% (*v*/*v*) DMSO served as the solvent control. A mycelial plug (5 mm in diameter) of the wild-type strain B05.10 was then inoculated onto the PDA plates containing the indicated compounds and incubated at 22 °C. Colony diameters and areas were measured after 24 h and 48 h of incubation. Each treatment consisted of at least three technical replicates, and the entire experiment was performed independently three times.

### 2.4. Dose–Response Assay of B. cinerea to rac-GR24

To assess the sensitivity of *B. cinerea* to increasing concentrations of *rac*-GR24, stock solutions of *rac*-GR24 were first dissolved in DMSO to prepare stock solutions at 1 mM, 5 mM, 10 mM, and 20 mM. Each stock was added at 0.1% (*v*/*v*) to cooled PDA medium to yield final working concentrations of 1 μM, 5 μM, 10 μM, and 20 μM, respectively. PDA supplemented with 0.1% (*v*/*v*) DMSO served as the solvent control. A mycelial plug (5 mm diameter) of the wild-type *B. cinerea* strain B05.10 was inoculated onto the prepared plates and incubated at 22 °C. Colony diameters and areas were measured after 48 h of cultivation. Each treatment consisted of at least three technical replicates, and the entire experiment was performed independently three times.

### 2.5. Detection of the Effects of Different Stereoisomers of SLs and GR24 Analogues on the Growth of Botrytis cinerea and Sclerotinia sclerotiorum

To assess the responses of *B. cinerea* (wild-type strain B05.10) and *S. sclerotiorum* to different stereoisomers of SLs and the synthetic analogue, stock solutions of (+)5-deoxystrigol (5DS), (-)5-deoxystrigol (*ent*-5DS), (-)2′-epi-5-deoxystrigol (4-deoxyorobanchol, 4DO), (+)2′-epi-5-deoxystrigol (*ent*-4DO), GR24^5DS^, GR24*^ent^*^−5DS^, GR24^4DO^, and GR24*^ent^*^−4DO^ were first dissolved in DMSO to prepare stock solutions at 10 mM; *rac*-GR24 was similarly prepared at 20 mM. Each stock was added at 0.1% (*v*/*v*) to cooled PDA medium. PDA supplemented with 0.1% (*v*/*v*) DMSO served as the solvent control. Mycelial plugs (5 mm diameter) of each strain were inoculated onto the respective plates and incubated at 22 °C. Colony diameters were measured at specified time points post-inoculation: 40 h for *B. cinerea* and 22 h for *S. sclerotiorum*. Each treatment consisted of at least three technical replicates, and the entire experiment was performed independently three times. To date, two major types of natural SLs have been isolated and characterized from plants: 4DO (representative of the orobanchol type and 5DS (representative of the strigol type). The chemical synthesis of the enantiomers *ent*-5DS and *ent*-4DO has been achieved through stereoselective synthetic strategies [40,55,56]. As the synthetic analogue that most closely recapitulates both the structural and functional attributes of natural SLs, GR24 also possesses four distinct stereoisomers owing to its unique stereochemical configuration: GR24^5DS^, GR24*^ent^*^-5DS^, GR24^4DO^, and GR24*^ent^*^-4DO^. Among these, GR24^5DS^ and GR24*^ent^*^-5DS^ are combined in equal proportions to form the commonly employed racemic mixture, *rac*-GR24, which is widely used in physiological and functional studies of SL signalling [57].

### 2.6. Detection of Effects of Two Stereoisomers in rac-GR24 on the Growth of Botrytis cinerea

To determine the contribution of the two stereoisomers present in rac-GR24 (GR24^5DS^ and GR24*^ent^*^-5DS^) to growth inhibition of *B. cinerea*, each compound was independently dissolved in DMSO to prepare stock solutions at 10 mM or 20 mM. For combination treatments, GR24^5DS^ and GR24*^ent^*^-5DS^ were added together to cooled PDA medium at 0.05% (*v*/*v*) each (total 0.1%, *v*/*v*), yielding final working concentrations of 5 μM + 5 μM or 10 μM + 10 μM, respectively. For comparison, *rac*-GR24 was added alone at 0.1% (*v*/*v*) to achieve final concentrations of 10 μM or 20 μM. PDA supplemented with 0.1% (*v*/*v*) DMSO served as the solvent control. Mycelial plugs (5 mm diameter) of the wild-type *B. cinerea* strain B05.10 were inoculated onto the prepared plates and incubated at 22 °C in the dark for 40 h. Colony diameters were measured at specified time points after inoculation. Each treatment consisted of at least three technical replicates, and the entire experiment was performed independently three times.

### 2.7. Quantification and Statistical Analysis

Each experiment included three biological replicates (*n* = 3), with three technical replicates per biological replicate. Two-group comparisons were analyzed using Student’s *t*-test, and multiple comparisons were assessed using one-way ANOVA followed by Tukey’s HSD post hoc test. Significance levels were calculated using GraphPad Prism 9.0. Different letters in the figures indicate significant differences at *p* < 0.01. In addition, ‘****’ denote the significance levels *p* < 0.0001.

## 3. Results

### 3.1. rac-GR24, a Synthetic SL Analogue, Inhibits the Growth of Botrytis cinerea

**Figure 1:**
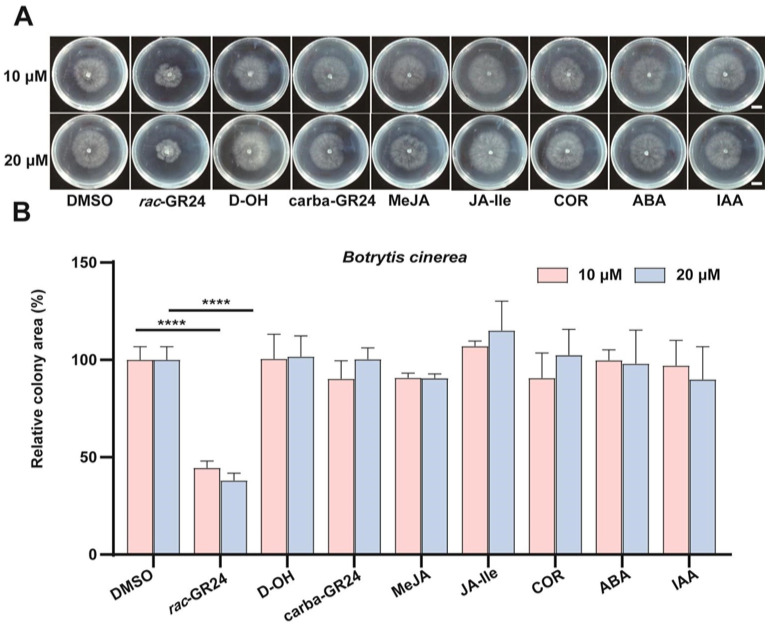
The synthetic strigolactone analogue GR24 inhibits the growth of *B. cinerea*. Growth status of the wild-type *B. cinerea* strain B05.10 on PDA medium supplemented with 10 or 20 μM of various plant hormones or their analogues after 48 h (**A**), scale bar = 10 mm; and quantification of the relative colony area ((**B**); mean ± SD, *n* = 3). ‘****’ indicate significant differences (*p* < 0.0001, two-tailed Student’s *t*-test).

To evaluate the effects of selected plant hormones and their analogues on the growth of *B. cinerea*, the wild-type strain B05.10 was treated with eight compounds (Figure S2) at two different concentrations, and subsequent colony growth was monitored and quantified. After 24 h of growth on medium supplemented with the respective compounds, only *rac*-GR24 significantly inhibited fungal growth compared with the DMSO control. The relative colony area in the *rac*-GR24 treatment group was 40–45% of that in the control group (Figure S1). Similar results were observed after 48 h of cultivation (Figure 1). These findings indicate that the synthetic SL analogue *rac*-GR24 exerts a significant inhibitory effect on the growth of *B. cinerea*. This is consistent with prior findings showing that GR24 suppresses the growth of multiple pathogenic fungi [48].

### 3.2. rac-GR24 Inhibits the Growth of Botrytis cinerea in a Concentration-Dependent Manner

**Figure 2:**
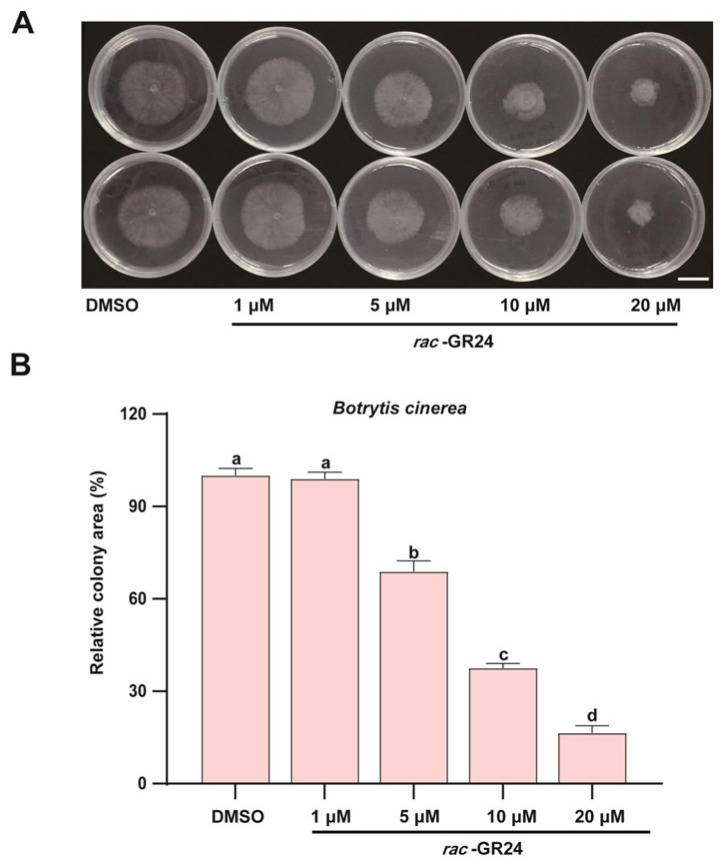
Effects of different concentrations of exogenous *rac*-GR24 on the growth of *B. cinerea*. Growth of the wild-type *B. cinerea* strain B05.10 cultured on PDA medium supplemented with the control (DMSO) or with 1, 5, 10, and 20 μM *rac*-GR24 for 48 h (**A**) and quantification of the relative colony area ((**B**); mean ± SD, *n* = 3), scale bar = 10 mm. Different letters indicate significant differences (*p* < 0.01, one-way ANOVA followed by Tukey’s HSD post hoc test).

To further characterize the inhibitory effect of the synthetic SL analogue *rac*-GR24 on *B. cinerea*, a refined concentration gradient of *rac*-GR24 was applied exogenously, and fungal growth was assessed. Following 48 h of treatment, significant inhibition was already observed at a concentration as low as 5 μM *rac*-GR24, with the colony area reduced to approximately 65–70% of that in the DMSO control group. Moreover, when the concentration of *rac*-GR24 was increased to 10 μM and 20 μM, a progressive reduction in colony area was observed under the same treatment duration, indicating an enhanced inhibitory effect with increasing concentrations (Figure 2). Collectively, these results demonstrate that *B. cinerea* growth is responsive to *rac*-GR24 even at low concentrations and is suppressed in a strictly concentration-dependent manner.

### 3.3. The Inhibitory Effects of Different GR24 and 5DS Stereoisomers on Botrytis cinerea Vary

**Figure 3:**
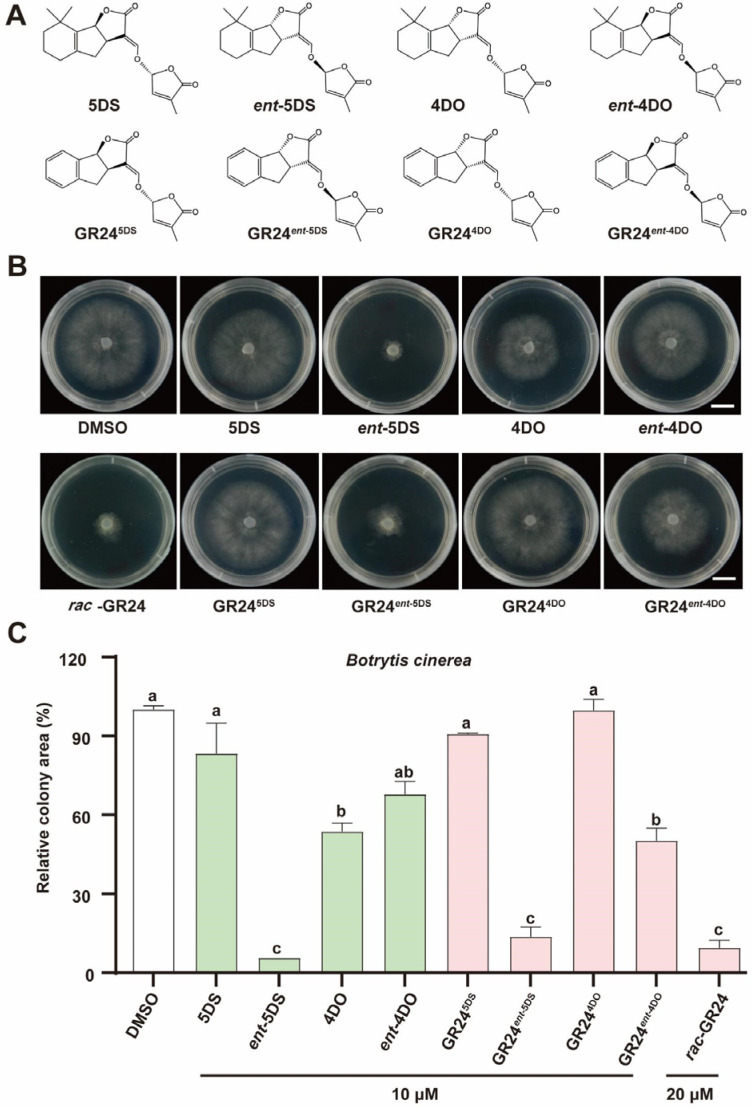
Effects of stereoisomers of strigolactones and the synthetic analogue GR24 on the growth of *B. cinerea*. Chemical structures of SLs or GR24 stereoisomers with different stereochemical features (**A**). Growth status of wild-type *B. cinerea* strain B05.10 after 40 h of treatment with DMSO (control), 10 μM of each of the eight stereoisomers derived from strigolactones and the synthetic analogue GR24, or 20 μM of the racemic mixture *rac*-GR24 (**B**), scale bar = 10 mm. The relative colony area were quantified ((**C**), mean ± SD, *n* = 3). Different letters indicate significant differences (*p* < 0.01, one-way ANOVA followed by Tukey’s HSD post hoc test).

To determine which stereoisomeric configuration of SLs predominantly accounts for the growth-regulatory effects on *B. cinerea*, we evaluated the antifungal activity of four corresponding stereoisomers. Notably, among the four SL-related stereoisomers examined, *ent*-5DS exerted a pronounced inhibitory effect on *B. cinerea*, reducing the colony area to less than 10% of that in the DMSO control group. In contrast, the other three compounds did not exhibit substantial suppression of fungal growth under the same experimental conditions (Figure 3).

To further determine whether the four stereoisomers of GR24 exhibit antifungal activity comparable to that of natural SLs, we conducted parallel experiments using each of the four GR24 stereoisomers as individual treatments. The results revealed that, analogous to the observations with natural SL enantiomers, the GR24*^ent^*^-5DS^ stereoisomer and racemic mixture *rac*-GR24 including GR24*^ent^*^-5DS^ potently inhibited *B. cinerea* growth, whereas the remaining three stereoisomers did not confer substantial suppression under the same experimental conditions (Figure 3). Collectively, these findings indicate that among the stereoisomers examined, the *ent*-5DS and GR24*^ent^*^-5DS^ configurations play a predominant role in restraining *B. cinerea* growth. Moreover, these results underscore the stereoselective nature of *B. cinerea* responses to both natural SLs and their synthetic analogues. Building upon previous reports that GR24 inhibits fungal growth [48], the present study provides a novel and significant finding: the filamentous fungus *B. cinerea* does not respond indiscriminately to all stereoisomeric configurations of GR24. Instead, it exhibits marked stereospecificity, responding exclusively to the *ent*-5DS enantiomer.

### 3.4. The Same Chiral Stereoselectivity Exerts Inhibitory Effects on Sclerotinia sclerotiorum

**Figure 4:**
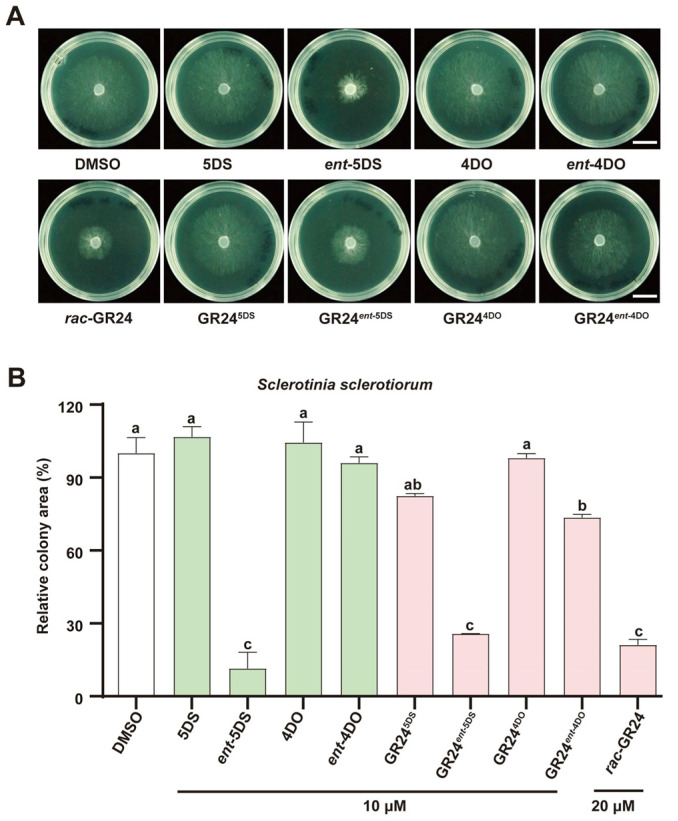
Effects of stereoisomers of strigolactones and the synthetic analogue GR24 on the growth of *S. sclerotiorum*. Growth status of wild-type *S. sclerotiorum* after treatment with DMSO (control), 10 μM of each of the eight stereoisomers derived from strigolactones and the synthetic analogue GR24, or 20 μM of the racemic mixture *rac*-GR24 (**A**), scale bar = 10 mm. The relative colony area were quantified ((**B**), mean ± SD, *n* = 3). Different letters indicate significant differences (*p* < 0.01, one-way ANOVA followed by Tukey’s HSD post hoc test).

To further validate the stereoisomeric selectivity of filamentous fungi toward this phytohormone and its analogues, parallel experiments were conducted using another necrotrophic fungal pathogen, *S. sclerotiorum*, employing the same panel of eight stereoisomeric compounds (Figure 4). Notably, *ent*-5DS, GR24*^ent^*^-5DS^ and the *rac*-GR24 mixture (which contains GR24*^ent^*^-5DS^) each exerted pronounced inhibitory effects on *S. sclerotiorum* growth (Figure 4). This pattern of stereoisomer-depending activity closely recapitulated the results observed in our previous experiments with *B. cinerea* (Figure 3). Collectively, these findings demonstrate that GR24 exhibits inhibitory activity against key filamentous fungi, with clear stereoselectivity. Furthermore, the consistency of the results across two distinct fungal species suggests that SLs and their synthetic analogue GR24 may exert broad spectrum inhibitory effects against filamentous fungi.

### 3.5. The GR24^ent-5DS^ Configuration Plays a Predominant Role in the Growth Inhibition of B. cinerea

**Figure 5:**
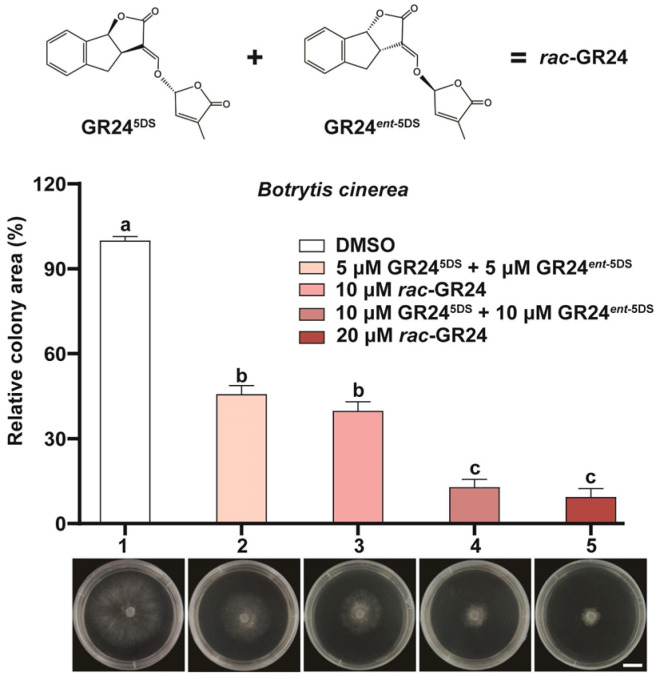
Effects of the mixture of GR24^5DS^ and GR24*^ent^*^-5DS^ on the growth of *B. cinerea*. The wild-type strain B05.10 of B. cinerea was treated with DMSO (1 in the upper panel, solvent control), two concentration combinations of GR245DS plus GR24ent-5DS (2 and 4 in the upper panel), or two concentrations of *rac*-GR24 (3 and 5 in the upper panel). Fungal colony growth on the culture medium was documented, and the relative colony area was quantified (Upper panel, mean ± SD, *n* = 3). Representative growth of *B. cinerea* corresponding to each treatment are shown in the lower panel below the x-axis (Lower panels); scale bar = 10 mm. Different letters above the bars indicate statistically significant differences (*p* < 0.01, one-way ANOVA followed by Tukey’s HSD post hoc test).

As described in the preceding experiments (Figure 3), the racemic mixture *rac*-GR24 consists of equal proportions of the two stereoisomers GR24^5DS^ and GR24*^ent^*^-5DS^ [57]. To further dissect whether the GR24*^ent^*^-5DS^ stereoisomer serves as the primary contributor to the growth suppressive activity of the mixture against *B. cinerea*, we performed a series of treatments using defined concentration combinations. Notably, the combination treatment elicited an inhibitory effect comparable to that of the double concentration racemic mixture, with nearly identical levels of growth suppression. To corroborate these findings, we applied a higher concentration combination consisting of 10 μM GR24^5DS^ plus 10 μM GR24*^ent^*^-5DS^ and compared its effect with that of 20 μM *rac*-GR24. Consistent with the results obtained at lower concentrations, both treatments exhibited equivalent inhibitory activity relative to the DMSO control (Figure 5).

## 4. Discussion

*B. cinerea,* as a devastating phytopathogenic fungus, inflicts substantial economic losses on agricultural production worldwide each year [1,11]. Recent reports have shown that the strigolactone (SL) analogue GR24 inhibits phytopathogenic fungi [48,58,59]. However, whether filamentous fungi possess a stereoselective recognition system for this compound and its natural counterparts remains unexplored. In this study, we demonstrate that *B. cinerea* and *S. sclerotiorum* exhibit a strict stereoselective response to both natural SLs and GR24, responding exclusively to the *ent*-5DS configuration (and its synthetic equivalent, GR24*^ent^*^-5DS^). In contrast, other stereoisomers, including 5DS, 4DO, and *ent*-4DO, as well as GR24^5DS^, showed no significant inhibition (Figure 1, Figure 2 and Figure 3, Figure S1). This finding extends beyond the mere antifungal activity of GR24 and reveals a previously uncharacterized level of chemical discrimination in fungi.

The observed stereospecificity is reminiscent of plant SL signalling, where the receptor D14 selectively recognizes the D-ring orientation of SLs, thereby triggering downstream developmental and symbiotic responses [60,61,62]. Notably, D14 hydrolyzes SLs and forms a covalent intermediate with the D-ring, a process highly sensitive to stereochemistry [50,57,63,64]. We speculate that a functionally analogous receptor may exist in filamentous fungi, capable of distinguishing the *ent*-5DS configuration. Notably, parallel experiments with *S. sclerotiorum* [2,17] revealed an analogous inhibitory profile (Figure 4), suggesting that GR24 exerts broad-spectrum and stereospecific antifungal activity. A widely used synthetic SL analogue, *rac*-GR24 consists of equal proportions of GR24^5DS^ and GR24*^ent^*^-5DS^ [57]. Our equimolar combination experiments showed that the inhibitory efficacy of the mixture is fully accounted for by GR24*^ent^*^-5DS^, with GR24^5DS^ contributing little additional activity (Figure 5). These data collectively demonstrate that the *ent*-5DS configuration is the main determinant of GR24’s antifungal activity against *B. cinerea*. The lack of activity of the other stereoisomers suggests that the putative fungal receptor imposes stringent structural requirements on the ligand, possibly due to a constrained ligand binding pocket or distinct hydrogen bonding networks. Future structural and molecular studies aimed at resolving the binding domain architecture of fungal versus plant receptors will be essential to explain the narrow stereochemical preference observed here.

Nevertheless, several limitations of this study should be acknowledged. First, although we have unequivocally demonstrated that *B. cinerea* and *S. sclerotiorum* exhibit strict stereoselectivity toward the *ent*-5DS configuration, the molecular basis of this recognition remains unknown. We have not identified the putative fungal receptor or effector proteins that directly bind GR24 or natural SLs. Without such target characterization, the causal link between stereochemical recognition and downstream growth inhibition remains possibility of correlation. Second, all experiments were conducted under in vitro conditions on synthetic media, which lack the complexity of the plant–fungus interface. Whether the same stereoselective inhibition occurs in planta—where plant-derived SLs, microbial competitors, and host immunity factors are present—has yet to be tested. Third, our study focused exclusively on vegetative growth; the potential effects of GR24 stereoisomers on fungal development, such as sclerotia formation, conidiation, or infection-related morphogenesis, were not examined. Finally, we did not perform genetic loss- or gain-of-function experiments to validate the proposed stereoselective pathway. Future efforts should integrate chemical proteomics, structural biology, and reverse genetics to isolate the fungal receptor for SLs. Moreover, in planta infection assays using stereoisomer-specific treatments, combined with transcriptomic or proteomic analyses, will be essential to decipher downstream signalling events and to evaluate the translational potential of enantioselective GR24 analogues as green fungicides. Despite these caveats, our findings establish a conceptual foundation for stereochemical targeting of fungal pathogens and open an avenue for rational design of next-generation crop protection agents.

## 5. Conclusions

In summary, through a screening of multiple plant hormones and their analogues, this study demonstrated that the synthetic SL analogue *rac*-GR24 effectively inhibits colony growth of *B. cinerea* with dose–response. Further stereochemical analysis revealed that both *B. cinerea* and another filamentous fungus *S. sclerotiorum* exhibit a strict stereoselective response, displaying strong inhibition exclusively toward the *ent*-5DS configuration, whereas other stereoisomers showed only marginal activity. Moreover, the antifungal activity of *rac*-GR24 was attributed predominantly to GR24*^ent^*^-5DS^, with GR24^5DS^ contributing minimally to the observed effect. Collectively, these findings provide physiological evidence for the stereoselective responsiveness of filamentous fungi to SL analogues.

## Data Availability

The original contributions presented in this study are included in the article/Supplementary Material. Further inquiries can be directed to the corresponding authors.

## Supplementary Materials

The following supporting information can be downloaded at: https://www.mdpi.com/article/10.3390/jof12050359/s1, Figure S1: The treatment of synthetic strigolactone analog GR24 inhibits the growth of *Botrytis cinerea* after 24 h treatment. Figure S2: Chemical structures of all compounds used in this study.
