# Supplementary material for: Stereospecific Antifungal Activity of Strigolactone Analogues Against Botrytis cinerea and Sclerotinia sclerotiorum
Source: J Fungi (Basel). 2026 May 13;12(5):359. doi: 10.3390/jof12050359 (PMC13209101; doi:10.3390/jof12050359)
Supplement: Supplementary file 1 [file jof-12-00359-s001.zip › jof-4271445-supplementary.pdf]

Supplementary Figure S1

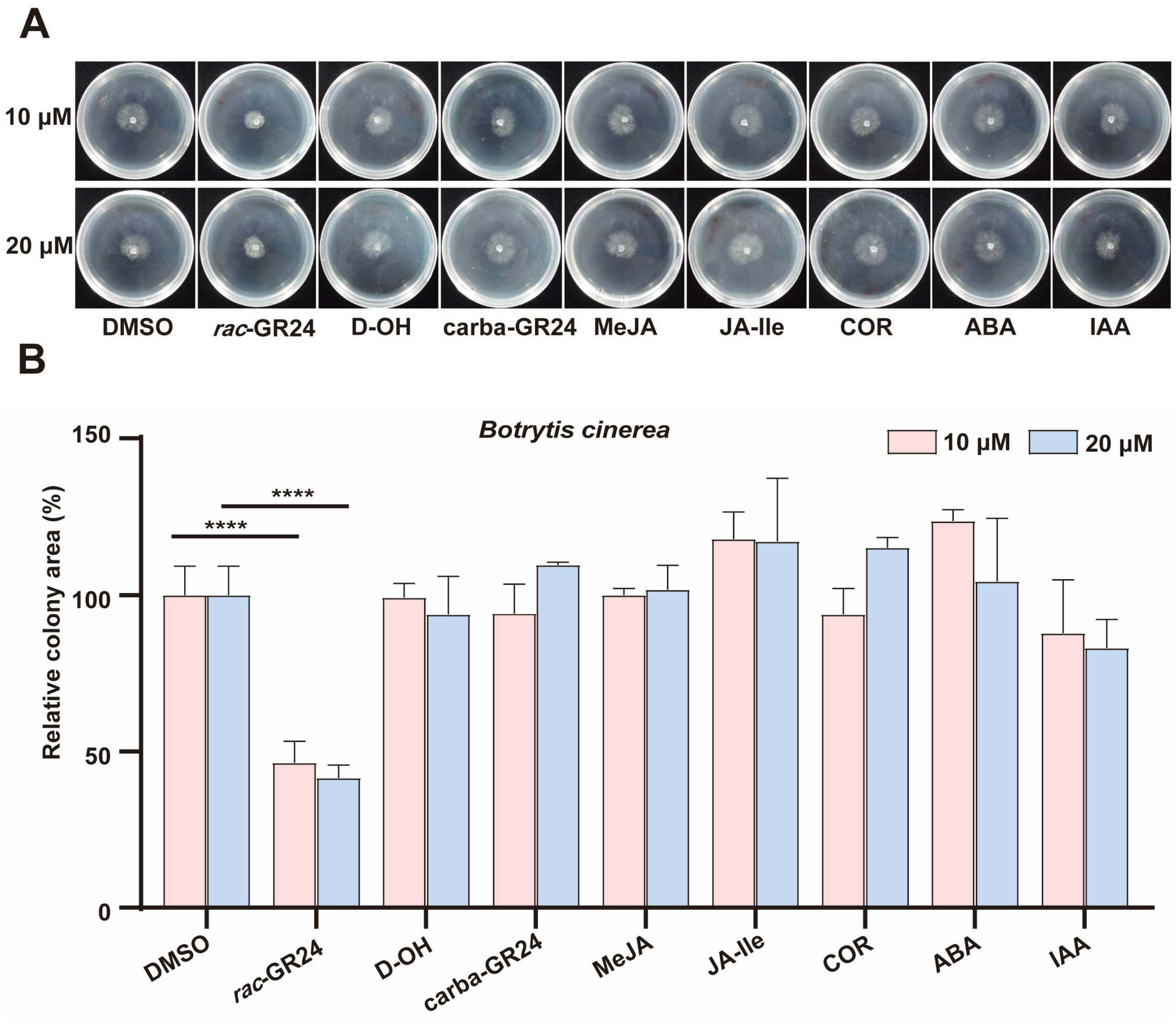

Figure S1. The treatment of synthetic strigolactone analog GR24 inhibits the growth of *B. cinerea* after 24 hours treatment.

Growth status of the wild-type *B. cinerea* strain B05.10 on PDA medium supplemented with 10 or 20 μM of various plant hormones or their analogues after 24 hours (A), scale bar = 10 mm; and quantification of the relative colony area (B; mean ± SD, n = 3). '****' indicate significant differences ($P < 0.0001$, two-tailed Student's t test).

Supplementary Figure S2

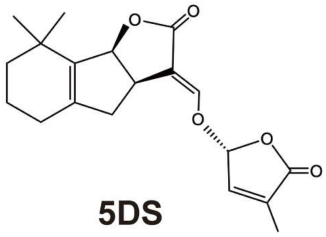

5DS

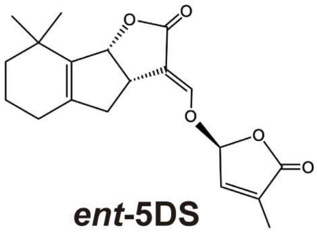

***ent*-5DS**

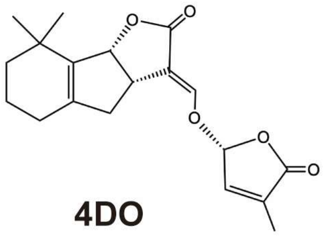

4DO

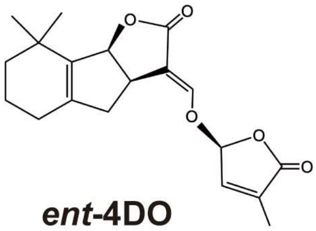

***ent*-4DO**

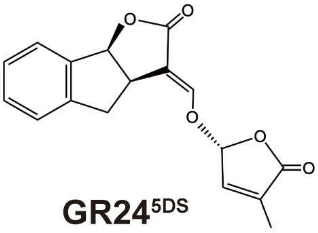

GR24^{5DS}

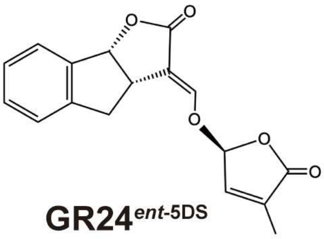

GR24$^{ent\text{-}5DS}$

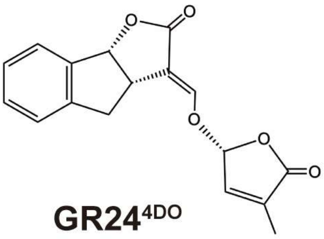

GR24^{4DO}

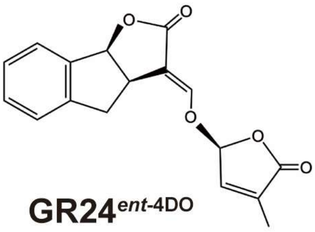

GR24$^{ent\text{-}4DO}$

***rac*-GR24**

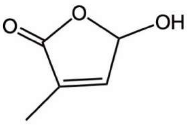

D-OH

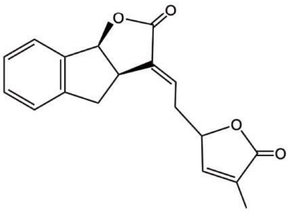

carba-GR24

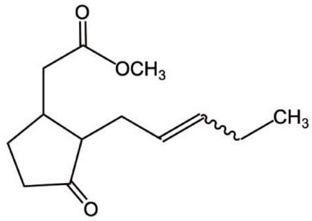

MeJA

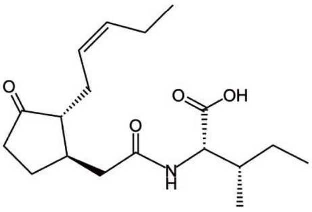

JA-Ile

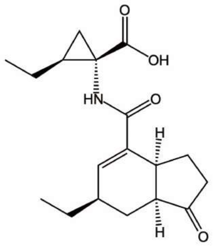

COR

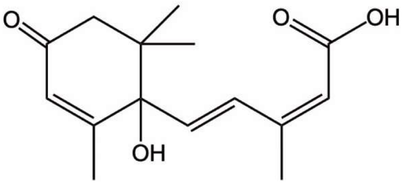

ABA

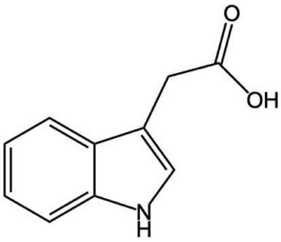

IAA

Figure S2. Chemical structures of all compounds used in this study.
